# Conserved language of plant gene regulation

**DOI:** 10.1093/plphys/kiaf263

**Published:** 2025-06-19

**Authors:** Neeta Lohani

**Affiliations:** Plant Physiology, American Society of Plant Biologists; Department of Biotechnology, Thapar Institute for Engineering and Technology, Patiala, Punjab 147004, India

Imagine your cell as a smart home where genes are like appliances that need to be turned on or off at the right times. This regulation is mediated by transcription factors (TFs), which act like intelligent operators that find and activate specific control switches (binding sites) near each gene to turn them on or off when conditions are right. TFs bind to specific DNA sequences called TF binding sites/motifs, which act like a molecular vocabulary—a limited set of “words” that cells use to communicate when and how genes should be expressed.

In *Arabidopsis*, over 1,500 TFs orchestrate the expression of tens of thousands of genes (Riechmann et al. 2000). Most of these TF families were already present in early land plants like the bryophyte *Marchantia* (Bowman et al. 2017). Bryophytes and flowering plants diverged from their common ancestor around 450 million years ago (Harris et al. 2022) (Figure. A). Since then, plant TF families have expanded and diversified, colonizing land and giving rise to hundreds of thousands of modern species. But here is the puzzle: as plant TF families expanded and diversified over hundreds of millions of years, what happened to their molecular vocabulary? Did TFs evolve new DNA recognition preferences to match their expanding regulatory roles, or did they maintain ancestral binding specificities while innovating in other ways?

**Figure.**
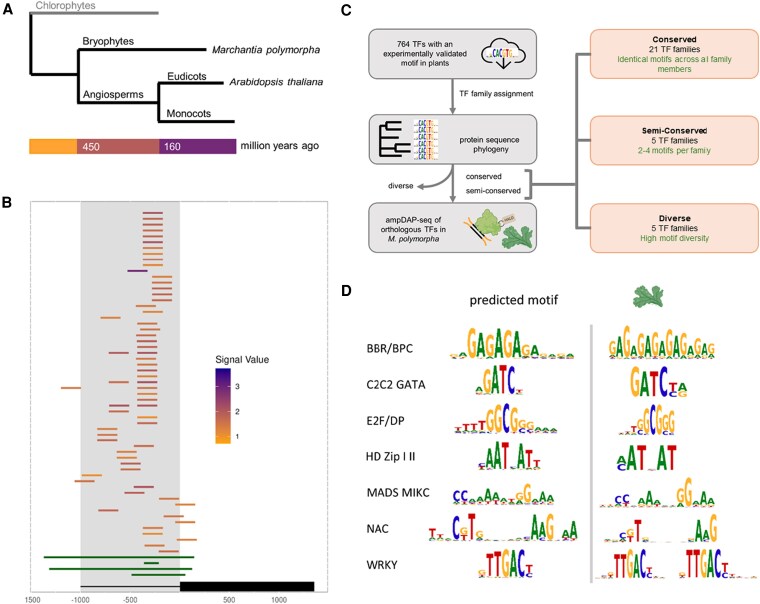
TFBM conservation analysis reveals 450-million-year evolutionary constraint in plant gene regulation. **A)** Phylogenetic relationships of major plant groups with available TFBM data and Chlorophytes (in grey) as an outgroup. Time scale indicates approximate divergence times, with the bryophyte-angiosperm split occurring ∼450 million years ago. **B)** Example of multiple TFs binding to the promoter of *CRF5* (*AT2G46310*). Each colored bar represents a different TF binding site, with stronger binding shown in purple and weaker binding in orange. Green bars show accessible DNA regions. The overlapping binding events demonstrate competition between TFs for the same regulatory sequences. **C)** TFBM analysis pipeline. 764 TFs with experimentally validated motifs were subjected to TF family assignment and phylogenetic analysis, resulting in classification into 3 evolutionary categories: conserved (21 families with identical motifs across all members), semi-conserved (5 families with 2 to 4 motifs per family), and diverse (5 families with high motif diversity). Predictions were validated through amplified DNA affinity purification sequencing experiments using orthologous TFs in *M. polymorpha*. **D)** Experimental validation of motif conservation across 450 million years of evolution. Sequence logos show predicted motifs from *Arabidopsis thaliana* (left) compared to experimentally determined motifs from *Marchantia polymorpha* (right) for 7 TF families. High similarity between bryophyte and angiosperm motifs demonstrates remarkable evolutionary conservation of DNA-binding specificities across major plant lineages.

In a recent article published in *Plant Physiology*, Zenker et al. 2025 provide insights into these questions and investigate how plant TFs have evolved their DNA-binding specificities over deep evolutionary time by analyzing binding data from hundreds of TFs across multiple plant species. The authors examined binding data for 686 *Arabidopsis* TFs across 50 TF families using published amplified DNA affinity purification sequencing data, which captures direct protein-DNA interactions without the confounding effects of chromatin structure by removing native DNA methylation (O’Malley et al. 2016; López-Vidriero et al. 2021). The authors mapped the binding patterns of these TFs across 27,206 gene promoters (1 kb upstream region from the transcription start site) and found 0 to 357 binding events per promoter (with one outlier showing 1,076 events). As demonstrated by the *CRF5* gene promoter (*AT2G46310*), the large positional overlap for TF binding sites provides evidence that multiple TFs bind to the same promoter regions (Figure. B). Next, to systematically analyze Transcription Factor Binding Motif (TFBM) conservation across plant evolution, the authors gathered 2,190 redundant TFBMs from public databases (JASPAR, Plant Cistrome, PlantTFDB). After removing redundant and inferred entries, 764 nonredundant TFs (686 from *Arabidopsis* and 78 from other species) from 13 plant species spanning 50 TF families remained for analysis (Figure. C). The authors found only 74 different consensus binding motifs, ranging from 5 to 21 base pairs in length, with an average length of 8.9  base pairs, suggesting that plant transcriptional regulation operates with a highly constrained molecular vocabulary. Some of these motifs are family-specific, while others can be recognized by members of multiple TF families, creating potential competition for the same regulatory elements.

The TFs were grouped into families based on their DNA-binding domains, subjected to phylogenetic clustering, and then classified as conserved, semi-conserved, or diverse depending on TFBM diversity within each family (Figure. C). The authors identified 21 conserved TF families that maintain identical binding preferences across all family members despite hundreds of millions of years of evolution. For example, the WRKY family, all 45 members, recognizes the same TTGAC sequence, and the GATA family members unanimously bind to GATC sequence. Interestingly, the TF family members sharing binding motifs often have overlapping expression patterns. This conservation severely limits the potential of conserved TF families for neofunctionalization through changes in DNA recognition.

The TF families containing 2 to 4 different binding motifs with no more than 15% outliers were classified as semi-conserved TF families (ARID, bHLH, bZIP, MYB, and MYB-related). Unlike conserved families where all members bind the same motif, these families are divided into subgroups with different binding preferences. The bHLH family binds to 2 distinct motifs, with functional specialization where members binding to the different motifs have distinct expression patterns within each subgroup, avoiding competition for DNA binding sites. The MYB-related family recognizes 3 different motifs corresponding to phylogenetic subclades, with some expression overlap within subgroups, creating competition among members binding the same motif. However, the bZIP and MYB families show extensive expression sharing among members binding the same motif. Additionally, experimental validation in *Marchantia* confirmed that these motif subgroups for the semi-conserved TFs have been conserved for at least 450 million years. Together, the semi-conserved TF families account for 18 of 74 total consensus motifs, showing reduced overall competition for binding sites compared to conserved families because competition is limited to subgroups rather than occurring among all family members.

Next, the authors identified 5 diverse TF families—ABI3/VP1, AP2/EREBP, C2H2, C3H, and Trihelix—with 5 or more different consensus binding motifs or more than 15% outliers, and together their 188 members cover 39 of the 74 total distinct binding motifs. The C2H2 zinc finger family leads this category with 13 different consensus binding motifs among 33 characterized members, including 32 from *Arabidopsis* and 1 from tomato. This diversity extends across plant species, with monocot TFs recognizing the same motifs as their most similar *Arabidopsis* orthologs, highlighting conservation of specific binding preferences within phylogenetic subgroups even in diverse families. Diverse TF families demonstrate that some TF groups readily evolve new binding motifs, creating possibilities for neofunctionalization through changes in DNA recognition.

Further to validate their observations, the authors performed experimental validation using the bryophyte *M. polymorpha* as a representative of early land plant evolution. Using DAP-seq on selected orthologous TFs from *Marchantia*, the authors experimentally validated their bioinformatic predictions about motif conservation. Representatives from conserved families yielded motifs identical to their flowering plant counterparts. For semi-conserved TF families, MYB-related representatives from all 3 subgroups (GATAA, GATATT, and TAGGG), and bHLH family representatives produced their expected subgroup-specific motifs. These findings demonstrate that TFBMs have remained stable for at least 450 million years.

This conservation creates a regulatory paradox: if TFs bind the same sequences, how do related TFs achieve different functions? The solution involves competition and context. TFs sharing binding motifs frequently have overlapping expression patterns, meaning they compete for identical regulatory sites. This competition explains why TF knockout experiments often produce mild effects—multiple factors can compensate for each other at shared binding sites (Zenker et al. 2025). These findings reveal that plants have primarily evolved through cis-regulatory changes (modifying promoter sequences) rather than trans-regulatory changes (evolving new TF binding specificities). This pattern holds for conserved and semi-conserved families, while diverse families readily evolve new binding motifs.

These findings open exciting new possibilities for improving crops. The stability of binding motifs across plant evolution suggests a promising strategy: manipulating the context in which existing TFs operate by adjusting their expression timing, modifying protein interactions, or altering binding site accessibility. Recent studies have demonstrated the efficiency of this approach. In rice and soybean, deleting repressor binding sites in NF-YC4 promoters led to increased leaf and seed protein content (Wang et al. 2025). Similarly, deleting the An-1 TF binding site in the IPA1 promoter enhanced rice yield by overcoming the trade-off between grain number and tiller number (Song et al. 2022). Advanced CRISPR-based promoter editing systems enable precise modifications to create beneficial trait variations (Zhou et al. 2023). Since binding motifs are conserved across plant species, successful modifications discovered in model plants can likely be transferred to major crops, potentially accelerating the development of climate-resilient varieties needed for sustainable agriculture.

## Data Availability

No new data were generated or analyzed in support of this article.
